# Comparison of anatomic axes with a navigated functional rotation axis determined by ligament tension for rotational femoral component alignment in cadaver knee arthroplasty

**DOI:** 10.1007/s00402-024-05394-3

**Published:** 2024-06-07

**Authors:** K Jäckle, M Pietzka, JP Schüttrumpf, B Panahi, M-P Meier, T Hawellek, W Lehmann, TA Walde

**Affiliations:** 1 Department of Trauma Surgery, Orthopaedics and Plastic Surgery, University Medical Center Göttingen, Robert-Koch Str. 40, 37075 Göttingen, Germany; 2 Department of Trauma Surgery, University of Magdeburg, Leipziger Str. 44, 39120 Magdeburg, Germany; 3 Radiology, Institute for Diagnostic and Interventional Radiology, University Medical Center Göttingen, Robert-Koch Str. 40, 37075 Göttingen, Germany

**Keywords:** Gonarthrosis, Navigation, Knee arthroplasty, Femoral malrotation, Ligament tension

## Abstract

**Introducion:**

The malimplantation of the total knee arthroplasty (TKA) components is one of the main reasons for revision surgery. For determining the correct intraoperative femoral rotation several anatomic rotational axes were described in order to achieve a parallel, balanced flexion gap. In this cadaveric study prevalent used rotational femoral axes and a navigated functional rotational axis were compared to the flexion-extension axis defined as the gold standard in rotation for femoral TKA component rotation.

**Materials and methods:**

Thirteen body donors with knee osteoarthritis (mean age: 78.85 ± 6.09; eight females and five males) were examined. Rotational computer tomography was performed on their lower extremities pre- and postoperatively. Knee joint arthroplasties were implanted and CT diagnostics were used to compare the preoperatively determined flexion-extension axis (FEA). The FEA is the axis determined by our surgical technique and serves as an internal reference. It was compared to other axes such as (i) the anatomical transepicondylar axis (aTEA), (ii) the surgical transepicondylar axis (sTEA), (iii) the posterior condylar axis (PCA) and (iv) the functional rotation axis (fRA).

**Results:**

Examination of 26 knee joint arthroplasties revealed a significant angular deviation (p^***^ < 0.0001) for all axes when the individual axes and FEA were compared. aTEA show mean angular deviation of 5.2° (± 4.5), sTEA was 2.7° (± 2.2), PCA 2.9° (± 2.3) and the deviation of fRA was 4.3° (± 2.7). A tendency towards external rotation was observed for the relative and maximum axis deviations of the aTEA to the FEA, for the sTEA and the fRA. However, the rotation of the posterior condylar axis was towards inwards.

**Conclusions:**

All axes showed a significant angular deviation from the FEA. We conclude that the presented technique achieves comparable results in terms of FEA reconstruction when compared with the use of the known surrogate axes, with certain deviations in terms of outliers in the internal or external rotation.

**Supplementary Information:**

The online version contains supplementary material available at 10.1007/s00402-024-05394-3.

## Introduction

The most frequently used procedure in the management of end-stage knee osteoarthritis is a total knee arthroplasty. Measured by the resulting pain relief and improvement in mobility, implantation of a total knee arthroplasty is a successful operation. However, malimplantation of the total knee arthroplasty (TKA) is one of the main reasons for revision surgery. Also, rotational malpositioning of the femoral component is often causing arthroplasty failures which, in turn, result in increased unilateral tension of the collateral ligamentous apparatus with consecutive contralateral instability. Due to the increased ligamentous tension, painful reduced flexion of the operated knee may occur [1–3]. In addition, there is a risk of painful patellar tracking up to subluxations [4]. The above-mentioned factors could result in reduced patient satisfaction which is directly related with joint function [5]. In particular, the unilateral increased ligament tension can cause increased pressure and shear stress on the polyethylene inlay. The consequence is the development of increased polyethylene abrasion, which subsequently can lead to aseptic component loosening which then needs revision surgery. One of the main goals in total knee arthroplasty is therefore to establish a parallel ligament-balanced flexion gap for mechanical alignment [6]. Several anatomical axes for intraoperative determination of femoral rotational alignment and a navigated functional rotation axis have been described in this context [7, 8]. In this regard the navigated functional rotation axis has the advantage that no additional ligament balancing is needed in order to achieve a parallel, ligament balanced flexion gap [8].

We performed a cadaveric study to compare different axes for intraoperative rotational alignment of the femoral component in knee arthroplasty. The anatomical axes and the functional ligament-tension-determined rotation axes (fRA) were compared with respect to the position of the flexion-extension axis (FEA). We report that the FEA is the optimal axis for femoral rotational alignment and can be easily determined by CT, although it cannot be determined intraoperatively [9]. The results suggest that an accurate and reliable measurement of the anatomical axes is relevant to avoid a malpositioning of the femoral prosthetic component, thus reducing a potential cause of implant failure. This study is intended to provide a direct input into clinical practice to assist total knee arthroplasty surgery.

## Materials and methods

### Cadaveric collective

The present study was approved by the ethics committee of the University Medical Center Göttingen (approval number: AN 24/7/13) and was in compliance with the Helsinki Declaration.

The cadaver collective consisted of a total of 13 body donors with bilateral primary knee osteoarthritis (mean age: 78.85 ± 6.09; eight females and five males; see Table 1), that underwent surgery. These numbers result in a total of 26 TKAs that were analyzed in the study. Each body donor had given the consent to the postmortem donation for medical studies during their lifetime.

**Table 1:** Baseline characteristics of the cadaveric collective

**Description**			
Number of body donors	13		
Age range [years]	69–89		
Age mean [years] ± SD	78.85 ± 6.09		
Body weight [kg] ± SD	78.08 ± 13.14		
**Gender**			
Female [n]	8		
Male [n]	5		
**Kellgren-Lawrence Classification**		**Knee side**	
***Body Donor [#]***		right	left
1		2	1
2		2	2
3		4	3
4		1	2
5		2	2
6		3	2
7		2	1
8		1	3
9		2	1
10		2	4
11		2	2
12		3	1
13		2	4
Mean ± SD		2.15 ± 0.80	2.15 ± 1.07
Mean female [*n* = 8] ± SD		1.88 ± 0.64	2.25 ± 1.28
Mean male [*n* = 5] ± SD		2.60 ± 0.89	2.00 ± 0.71

The body donors were evaluated before they became part of the study with regard to the exclusion criteria defined before the start of the study as listed below. The selection for the study was done shortly after the death of the body donors so that a maximum of one week elapsed between death and implantation. During this time the bodies were stored at 4 °C.

Exclusion criteria were defined as clinically uncorrectable deviations in the coronal plane in the sense of severe extraarticular varus or valgus malalignment. The presence of a total knee joint arthroplasty and clinically reduced mobility in the sense of arthrofibrosis or joint stiffness were additional characteristics decisive for exclusion.

### Pre- and postoperative computer tomography (CT)

Each cadaver underwent preoperative and postoperative rotational computer tomography (CT) of the lower extremities. Three acquisition points (hip, knee, ankle) were defined in the scout view of the spiral CT (Somatom, Definition Flash 2 × 64 lines DE, Erlangen, Germany). For each examination level, 0.75 mm slices and transverse, coronal, and sagittal multiplanar reconstructions were acquired in the bone algorithm in 2 mm slices. Positioning and fixation of the lower extremity for CT diagnostic was performed in a neutral zero position. It means the cadavers were positioned in supine position on the CT table and the knee was stretched as much as possible. To avoid measurement errors due to different preoperative and postoperative positioning of the lower extremity, a Kirschner wire (K-wire) was inserted into the cadavers as a reference axis in the femoral shaft before the initial preoperative CT scan. This ensured an exact determination and comparison of the respective axes pre- and postoperatively. Based on the preoperative CT scan or scout view, the degree of osteoarthritis was determined according to the Kellgren and Lawrence classification (see Table 1).

A scout view was obtained, and multislice CT scans of the knees were taken from the femoral distal metaphysis perpendicular to mechanical axes of the femur and examined by an experienced radiologist. The rotation of the distal femur was evaluated using single axial CT images through the femoral epicondyles. The lines of the anatomical axes were drawn digitally in this view on the monitor. Then the radiologist measured the angles along these lines digitally on the monitor (see Table 2).

**Table 2:** Overview of the significances of the absolute mean angular deviations

Description	Mean axis deviation [°]	Range axis deviation [°]	Significance *p*
aTEA to FEA vs. sTEA to FEA	2.51	0.28 to 4.73	0.036 (*)
aTEA to FEA vs. PCA to FEA	2.24	0.01 to 4.47	0.028 (*)
aTEA to FEA vs. fRA to FEA	0.96	-1.27 to 3.19	0.059 (n.s.)
sTEA to FEA vs. PCA to FEA	0.27	-2.49 to 1.96	0.072 (n.s.)
sTEA to FEA vs. fRA to FEA	1.54	-3.77 to 0.69	0.068 (n.s.)
PCA to FEA vs. fRA to FEA	1.28	-3.5 to 0.95	0.054 (n.s.)

aTEA = anatomical transepicondylar axis; FEA = flexion-extension axis; sTEA = surgical transepicondylar axis; PCA = posterior condylar axis; fRA = functional rotation axis; n.s. = not significant; * = statistically significant

### Surgical technology

Implantation of the total knee joint arthroplasties was performed using the navigated technique with the PiGalileo TKR light® navigation system (Smith & Nephew GmbH; Hamburg, Germany). This imageless system supports the surgeon in aligning the individual components using bony landmarks and various axes of the knee joint. The anatomic landmarks used were (i) the most prominent points of the medial and lateral epicondyles and (ii) the anterior sulcus (Whitesides line). Stationary reference locators were positioned on the tibia and femur at the beginning of the operation. A mobile locator was also used to determine bony landmarks. The locators are each equipped with reflector balls. In addition, intraoperative data transmission was performed with an infrared camera connected to a computer. The accuracy of the navigation system is also specified in the manufacturer’s operating instructions as ± 2 mm (operating instructions PiGalileo TKR Light® 2006).

First, the standard surgical approach to the knee joint was performed by means of a midline incision. After preparation of the subcutaneous tissue, the knee joint was opened *via* a parapatellar medial arthrotomy. While sparing the vastus medialis muscle, the incision was then extended proximally in the quadriceps tendon and distally to the medial tibial tuberosity, thus allowing the patella to be laterally everted. Next, the stationary femoral reference locator was placed via the described approach on the ventral distal femur. The tibial reference locator was then attached to the distal tibia by means of two, small stab incisions. Next, the limb was registered electronically starting with the recording of the femur. Next, the center of rotation of the hip was determined. Determination of the mechanical femoral axis was completed by registering the distal piercing point using a mobile reference locator. Subsequently, bony reference points on the distal femur are successively worked through by the mobile reference locator according to the manufacturer’s protocol (PiGalileo TKR Light® 2006) and registered by the navigation system. The following bony landmarks were used to determine the resection depth of the distal femur: (i) distal shaft center (femoral notch), (ii) posterior medial and lateral condyles, (iii) medial and lateral epicondyles, (iv) distal lateral and medial condyles.

Afterwards, the mobile reference locator was used to register bone reference points on the tibia, which are the distal and proximal tibial center as well as the mediolateral and anteroposterior border of the tibial plateau. To determine the mechanical tibial axis, the central point in the area of the eminentia intercondylaris was registered first. Registration was followed by looking for the deepest points of the medial and lateral articular surfaces for subsequent determination of the tibial resection depth. The tibial rotation was then registered and recorded. For the final determination of the mechanical tibial axis, the distal fibular tip and the most prominent elevation of the medial malleolus were then registered by the mobile reference locator.

Implantation of the total knee joint arthroplasties was performed using the tibia-first technique. With this technique, the preparation of the tibial side was started after the registration had been completed. The tibial saw block was attached to the proximal tibia in a controlled manner using navigation. During the subsequent navigation-assisted final adjustment, the tibial slope, the tibial resection height and the valgus or varus deviations were displayed on the monitor. These technical aspects were corrected or readjusted in a controlled manner prior to the tibial bone resection. This procedure was performed by adjusting screws on the saw block according to the data from the navigation system. After the tibial bone resection had been performed in 90° for a mechanical alignment, the quality of the saw cut was registered by the navigation device. As with the tibia, the distal saw block was first positioned with the aid of navigation. After fine adjustment based on navigation, the distal femoral cut was made and registered by the navigation system in the same way as the tibial saw cut.

**Fig. 1:**
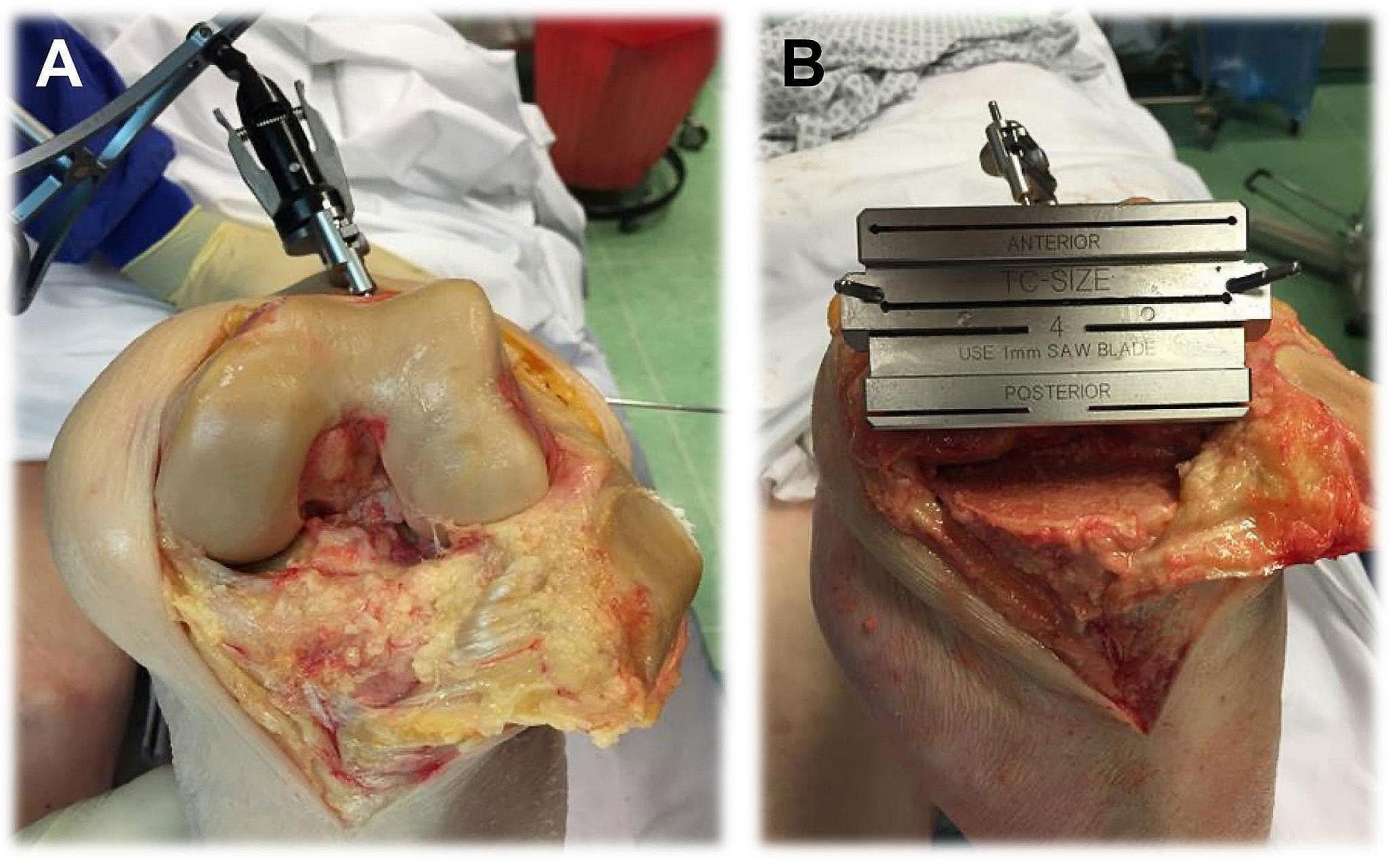
Cadaver preparation. (**A**) Femoral condyles after resection of the anterior cruciate ligament, note incision of the femoral reference locator in the upper image section. (**B**) Attached four-in-one saw block

The femoral rotation axis determined in the procedure described below is referred to as the functional rotation axis (fRA). The first step was to fix the femur size guide in 90° flexion of the knee joint. The posterior condyles on the distal femoral resection surface were used as an internal reference. The mobile reference locator was then attached to the femoral size guide. Accordingly, all changes with regard to femoral rotation could be recorded and registered by the navigation system. A strap tensioner was then inserted into the flexion gap and tensioned laterally with 60 N to 80 N. The medial tension was not determined. The femoral size gauge was then attached to the femur, and fine adjustment of the rotation and anterior-posterior positioning was then carried out by means of adjusting screws on the femur size gauge. Under these conditions, a rotation correction of internal/external rotation of up to 6° was possible. The target value here represents the parallelism to the tibial saw cut to achieve a parallel, band-balanced flexion gap. After fine adjustment of the sizing gauge, two holes were drilled for attaching the “four-in-one saw block” to place the saw block, to subsequently fix it, and the four resections could be made (see Fig. 1). Once the trial implants had been inserted, a new functional analysis was performed analogous to the preoperative functional analysis. Finally, the implants were removed again so that an artifact-free postoperative CT could be performed to accurately determine the functional rotation axis (fRA).

### Evaluation of the axes as a function of the flexion-extension axis

The anatomical axes were compared with the flexion-extension axis (FEA) using a preoperative and postoperative CT analysis. To ensure accurate and artifact-free measurements, the inserted prosthetic components were removed prior to performing the postoperative CT. Measurements were performed using Centricity PACS software (version 10.1; General Electrics Healthcare; Chicago, USA).

**Fig. 2:**
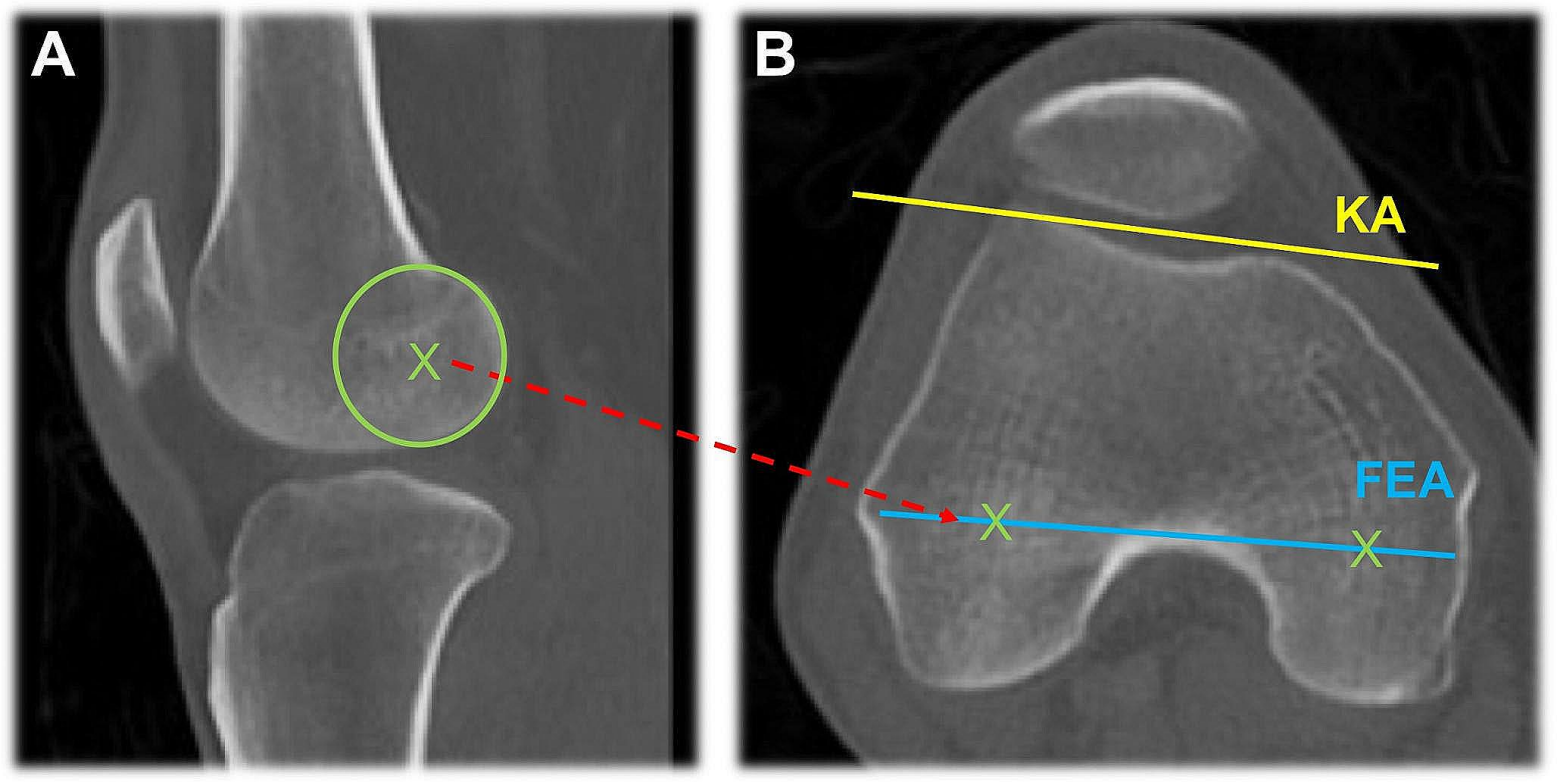
CT imaging of the knee. (**A**) Determination of the posterior centers of rotation marked with green “X” and (**B**) transfer to the axial section plane. X = posterior centers of rotation, KA = K-wire axis and FEA = flexion-extension axis. Areas refer to the bony structures

In preoperative CT, FEA was determined by establishing the centers of rotation of the medial and lateral femoral condyle. These parameters were examined in the respective sagittal section plane according to the largest anterior-posterior condyle diameter. Accordingly, the two centers of rotation could be transferred to the axial section plane using a corresponding objective function of the Centricity PACS software. This way the angle to the K-wire axis (KA) could be determined (see Fig. 2). The KA was defined by a Kirschner wire which was inserted in both distal femora. Thus, it could serve as a reference axis of comparability of the pre- and postoperative data.

**Fig. 3:**
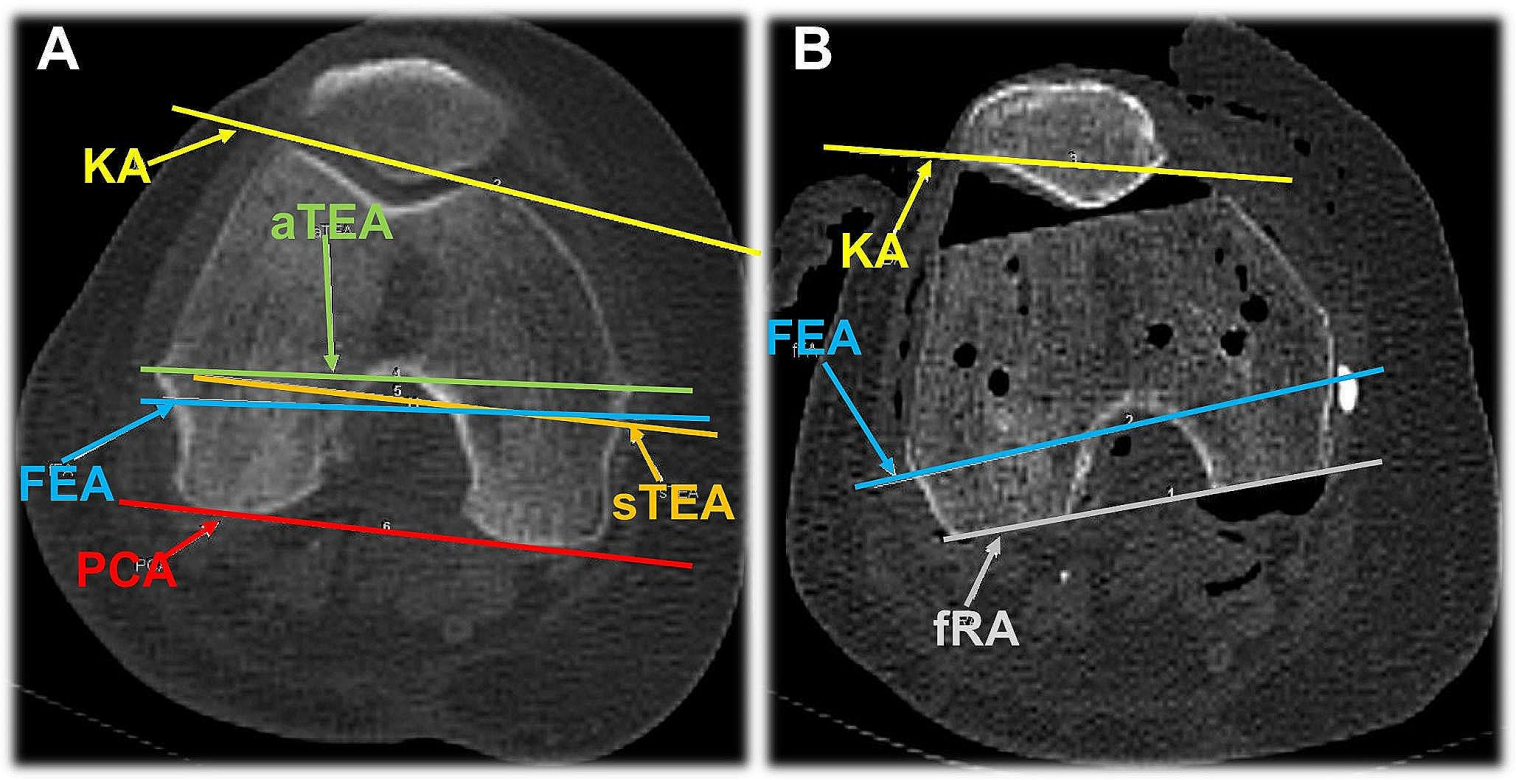
Axial CT imaging of the knee. (**A**) Preoperative determination of anatomic axes. KA = K-wire axis, aTEA = anatomic transepicondylar axis, sTEA = surgical transepicondylar axis, FEA = flexion-extension axis, and PCA = posterior condylar axis. (**B**) Postoperative axis determination. KA = K-wire axis, FEA = flexion-extension axis and fRA = functional rotation axis

To measure the individual angles, an axial section plane was first determined in which the anatomical axes (aTEA, sTEA, PCA) could be drawn according to their bony landmarks. For comparison, the FEA had to be transferred to the respective section plane according to its angle to the K-wire axis and drawn in. In the preoperative CT, the individual angles of the aTEA, sTEA, PCA and FEA to this axis were determined (see Fig. 3A).

The functional axis of rotation (fRA) was determined on postoperative CT imaging (see Fig. 3B), corresponding to the dorsal resection area on the femur. In addition, the angle to the K-wire axis (KA) was measured (see Fig. 3). After determining the individual axes or their angles to each other, the axes of the aTEA, sTEA, PCA and the fRA were compared to the flexion-extension axis (FEA) which represents the physiological flexion-extension axis that was set equal to zero. In addition, for axis deviations into external rotation, the respective angle was marked by a positive sign, while for axis deviations into internal rotation, it was marked by a negative sign.

### Statistics

**Fig. 4:**
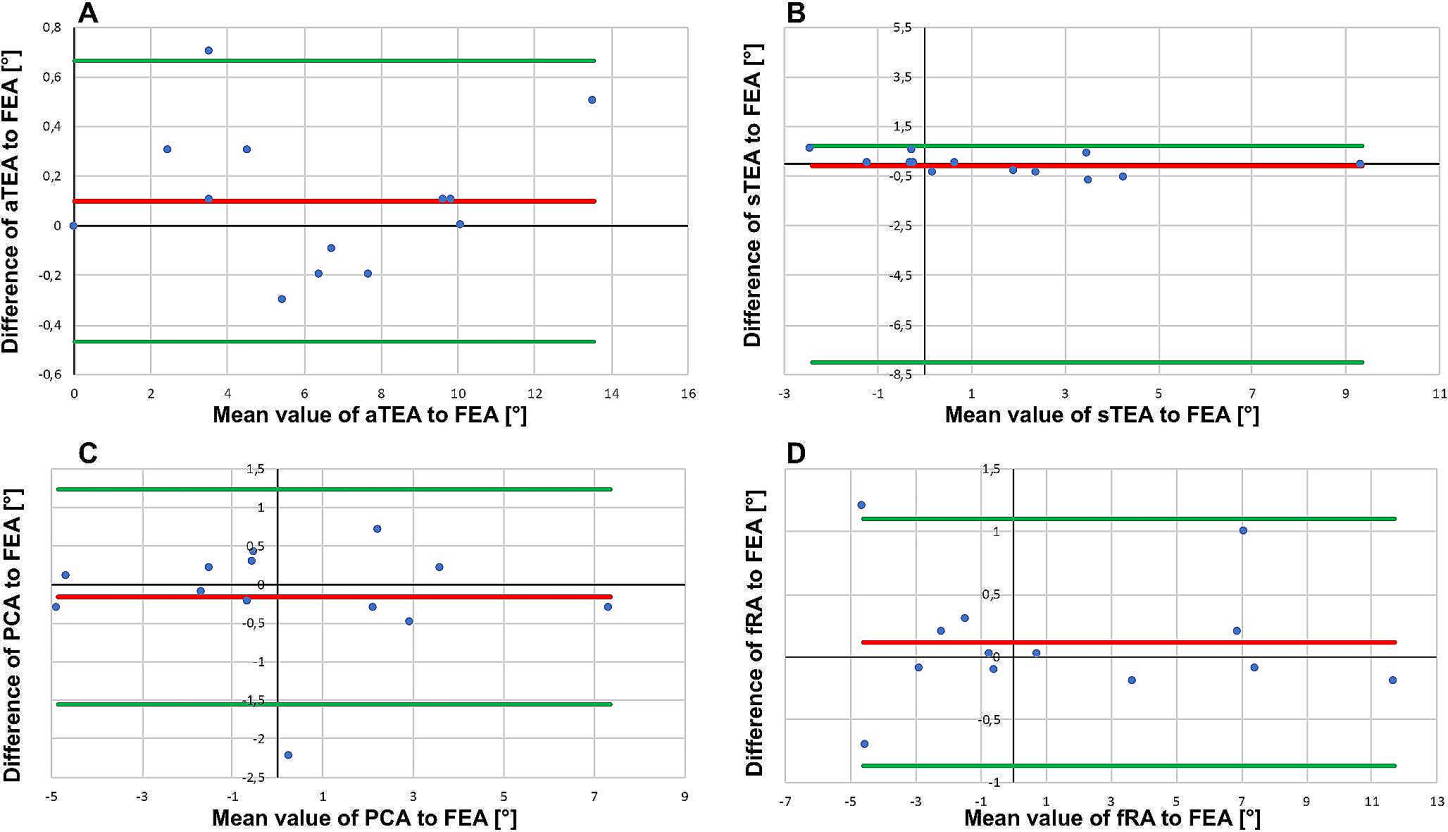
Bland-Altman plot to compare the different axes with the FEA. (**A**) Comparison of aTEA to FEA, sTEA to FEA (**B**), comparison of PCA to FEA (**C**) and comparison of fRA to FEA (**D**). aTEA = anatomic transepicondylar axis, sTEA = surgical transepicondylar axis, FEA = flexion-extension axis, PCA = posterior condylar axis and fRA = functional rotation axis. Difference of the different axes to FEA in degree (°) shown on the y-axis, mean value of the different axes to FEA in degree (°) shown on the x-axis. The mean value of the differences is shown as a continuous red line. The green lines represent the mean value of the differences ± 1.96 standard deviation of the differences

Statistical analysis was performed using the D’Agostino-Pearson, Kolmogorov-Smirnov and Shapiro-Wilk tests to test for normal distribution. The significance calculation was based on the sample t-test for normally distributed variables and the significance level was set to alpha = 5%. The data are presented as medians and interquartile ranges in box plots and as means and standard deviations. A Bland-Altman plot was created to compare the measurements of the different axes with the FEA (Fig. 4A-D). The test-retest reliability was determined using Cohens’s kappa κ for the interrater reliability. For all statistical tests, the statistics software GraphPad Prism (Version 5.04, GraphPad Software Inc.; San Diego, USA) was used.

## Results

According to Kellgren and Lawrence [10], six of the 26 operated knee joints were independently classified as grade 1, 13 cases grade 2, in 4 cases grade 3 and in 3 cases grade 4, respectively (see Table 1) by two different surgeons.

**Fig. 5:**
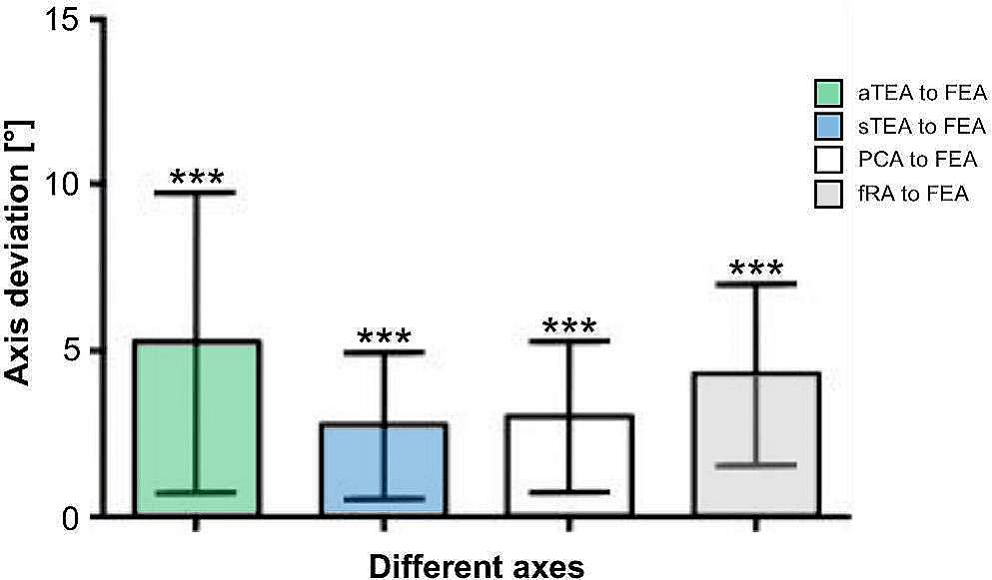
Absolute angular deviation of the individual axes from the FEA. Representation of absolute angular deviation of each axis compared to FEA (*n* = 26). Axis deviation in degree (°) shown on the y-axis, localization of different axes shown on the x-axis. Error bars represent the standard deviation, *** = < 0.0001

The statistical evaluation showed a significant angular deviation in external direction for all axes (p^***^ < 0.0001) in reference to FEA. In case of aTEA an absolute mean angular deviation of 5.2° (± 4.5) was found, in case of sTEA the deviation was 2.7° (± 2.2), with PCA 2.9° (± 2.3) and, finally, the deviation of fRA was 4.3° (± 2.7) (see Fig. 5).

Twenty-two of the knee joints (84.6%) showed a relative external axis deviation of the aTEA to the FEA (mean value: 5.7° ± 3.8). Considering the relative axial deviation of the sTEA, a substantial fraction of the knee joints (*n* = 11; 42.3%) showed a tendency toward external rotation (mean value: 4.1° ± 2.1). This finding indicates a clear tendency of both the aTEA and sTEA axes toward an external rotation.

In contrast, the relative axis deviation of the PCA to the FEA of the knee joints shows an internal rotation (*n* = 12; 46.2%) with a mean value of 3.9° ± 2.4, but in 5 knee joints (19.2%) there was no deviation of the PCA to the FEA (mean value: 0.6° ± 0.3) from this axis. This result indicates that there is a tendency of the axis deviation of the PCA to internal rotation. Furthermore, for 13 knee joints (50%), the relative axis deviation from the fRA to the FEA results in an external rotation (mean value: 5.9° ± 2.3). The maximum axis deviation of aTEA to FEA was found to be 13.8° (external rotation) and 7.2° (internal rotation), respectively. The sTEA also shows a maximum deviation of 9.3° (external rotation) and 5.2° (the internal rotation). Furthermore, the maximum axis deviation of the PCA to FEA is 7.2° (external rotation) and 10.7° (internal rotation), respectively. In addition, the fRA to FEA has a maximum axis deviation of 11.6° (external rotation) and of 5.8° (internal rotation).

The Bland-Altman plots show a distribution of values within the fluctuation range, with the exception of a few outliers (see Fig. 4A-D). The interrater reliability was also calculated and resulted in a Cohen’s Kappa value of 0.591 for the comparison of the fRA to the FEA. This result indicates a moderate agreement between the measured values. For aTEA/FEA the Cohen’s Kappa κ value was 0.471 and thus, there is again a moderate agreement, whereas sTEA/FEA show a value of 0.191 and for PCA/FEA it was 0.189, which represents a low agreement.

## Discussion

Implantation of a total knee arthroplasty (TKA) is the most common surgical therapeutic option for the treatment of primary and secondary knee joint osteoarthritis after conservative therapeutic measures have failed [11]. In addition to periprosthetic infection and aseptic loosening, a malpositioning of prosthetic components is the main reasons for surgical revision of primary prosthetic implantation [1–3].

The malposition of the femoral prosthetic component is still a relevant cause of implant failure or clinical failure, i.e. massive pain symptoms after TKA [1–3]. Our study made use of CT-based data sets to compare the anatomic axes of the distal femur with the individual flexion-extension axis (FEA) and a rotation axis (fRA) as determined by ligament tension. After TKA, all axes showed a significant absolute mean angular deviation from FEA. Thus, a potentially clinically relevant false femoral rotation may occur in either internal or external rotation. However, the extent to which kinematic alignment objectively improves knee balance in total TKA is still unknown., MacDessi et al. [2] already addressed whether restoring constitutional alignment results in better quantitative knee balance than mechanical alignment. In their study, MacDessi et al. [2] investigated an optimal knee balance by measuring intercompartmental pressure differences using corresponding sensors. The results indicate that restoration of constitutional alignment in TKA leads to a statistically significant improvement in the quantitative balance of the knee. Furthermore, with regard to the correct rotational positioning of the prosthetic components, the appropriate rotational alignment of the femoral component represents a major aspect for the implantation of a total knee arthroplasty [2, 12].

As a reference for correct alignment of prosthetic components in the frontal plane, the long anatomical axes of femur and tibia bones are commonly used [13, 14]. A major goal of navigated total knee arthroplasty is to further improve implantation accuracy and reconstruct the physiological leg axis, especially in the frontal plane. This goal is intended to minimize a premature loosening of the prosthetic components. In this regard, improved alignment of prosthetic components using navigation has been documented in several earlier studies [13–15]. However, a study by Abdel et al. [16] also indicates that an increased axial deviation in the frontal plane does not significantly affect prosthesis longevity. As a consequence, the insertion of knee joint arthroplasties by navigation has seen a significant decline in recent years.

Incorrect ligamentary balancing of the knee joint can also lead to clinically relevant instabilities and incorrect loading of the artificial knee joint postoperatively and, in turn, to prosthesis failure with necessary revision surgery [17]. A symmetrically ligament-balanced extension and flexion gap is therefore one of the basic requirements for a well-functioning total knee joint arthroplasty as already eluted to by Lotke and Ecker [18]. Of particular importance is the rotational positioning of the tibial and especially the femoral component [1, 3]. With regard to femoral rotational alignment in the transverse plane, Walde et al. [8] have demonstrated that proper navigation reliably determines functional rotation of the femoral component. This rotation appears to be crucial for the interaction of the tibiofemoral and the patellofemoral joint. Although femoral rotation does not essentially influence the extension gap, it nevertheless plays an important role in the stability of the flexion gap [1–3]. A prerequisite for walking on uneven ground and standing up without pain is a stable, balanced flexion gap [19].

The clinical relevance of femoral malrotation is already explained in the study of Bell et al. [6]. In their study, they describe the rotational alignment of components in a cohort of 56 patients with unexplained pain after total knee arthroplasty with a matched control cohort. The results show that a misalignment of both internal rotation of the tibial and femoral components was found to be a significant factor in pain after TKA. These studies indicate that this aspect of knee arthroplasty is still very topical and important.

The FEA as a femoral rotation axis is the connection of the rotation centers of the medial and lateral femoral condyle. Thus, its spatial relationship to anatomic landmarks represents a key to total knee arthroplasty and it helps to plan the prosthesis design with respect to the ligamentous alignment and balancing, respectively. Furthermore, current femoral prosthetic components often feature two symmetrical condyles and an identical sagittal curvature. The line connecting the respective dorsal condylar centers thus forms the flexion/extension center. After implantation of the femoral component, this line should ideally be congruent or parallel to the FEA [20]. According to Pagnano et al. [21], the sTEA can serve as a useful landmark for neutral rotational alignment of the femoral component. If the latter is positioned parallel to the sTEA, a lower incidence of “femoral lift-off” has been reported [22, 23]. In our study, the sTEA showed the smallest absolute deviation of 2.7° (± 2.2) from the FEA as compared to the other axes. However, as with the other axes, this was also statistically significant (p^***^ < 0.0001). The sTEA showed a tendency to external rotation deviation in 42.3% of the cases with a maximum of 9.3°. The maximum internal rotation deviation showed the lowest value of 5.2° compared to the other axes. Furthermore, the sTEA was statistically significantly different from the aTEA (p^*^ = 0.036), but not from the PCA (*p* = 0.072) and fRA (*p* = 0.085), respectively.

Our study suggests that the sTEA is characterized by a quite reliable determinant based on CT diagnostics. Based on these results of our study, the use of sTEA for femoral rotational alignment based on CT diagnostics can indeed be recommended. The development of surgical methods for reliable intraoperative detection of sTEA would be highly meaningful for clinical application. Large differences in rotational positioning of the femoral component, such as extreme external rotation positioning, occurred during alignment using aTEA. In our study, the aTEA of 5.2° (± 4.5) also showed a statistically significant absolute deviation from the FEA (p^***^ < 0.0001). This was the largest deviation when compared to the other axes. The results of our study are in accordance with the results obtained in the study of Kumar and Sharma (2018) [24]. In clinical application, the difficulty of reliable intraoperative detection of the bony reference points is therefore to be considered, since the clinically relevant femoral malrotations, as obtained with sTEA are expected. For this reason, aTEA can definitely not be recommended as a reliable reference point in primary total knee arthroplasty. Thus, the development of improved techniques for intraoperative determination is essential. In revision arthroplasty, however, aTEA and sTEA consolidate their position in the absence of applicability of other axes. As an alternative to femoral intraoperative rotational alignment, a ligament tension-adapted technique has been described [8]. With this technique, femoral rotation is determined by a ligament tensioner with reference to the tibial saw cut to create a parallel, ligament-balanced flexion gap. Here, we describe a specific ligament tension and rotational orientation parallel to the tibial saw cut compared to the traditional bony landmarks. This investigated feature of the FEA reconstruction achieves similar results when compared to the known equivalent axes.

Dennis et al. [25] succeeded in demonstrating a significantly lower incidence of “femoral lift-off” by alignment using a ligament-tension-balanced flexion gap. We observed a statistically significant absolute deviation from the FEA with 4.3° (± 2.7) for the fRA (p^***^ < 0.0001). The relative deviation to the FEA showed a tendency to femoral external rotation in 50% of the cases with a maximum deviation of 11.6°. Furthermore, femoral internal rotation occurred in 30.8% of the cases with a maximum deviation of 5.8°.

Due to the known intraoperative difficulty to correctly reconstruct femoral rotation, one of the current concerns is the correct soft tissue balancing of the collateral ligamentous apparatus [26]. The anatomical, bony reconstruction of the femoral rotation would thus become less important and techniques for reliable lateral ligament balancing, such as our described methodology, would again take precedence. The surgical technique described above with generation of the fRA achieves a parallel, laterally band-balanced flexion gap. Consequently, a mean absolute angular deviation of the fRA from the FEA of 4.3° or a relative internal rotation deviation in approximately 30% of cases must be accepted in favor of a ligament-balanced flexion gap. This appears to be an inherent problem of the surgical technique, since this internal rotation deviation cannot be explained otherwise. The occurrence of clinically relevant imbalances in the area of the ligamentous apparatus, as in the application of the anatomical axes, is then extremely unlikely.

Furthermore, the described ligament-tension-determined technique can not be recommended without reservation, since the intactness of the collateral ligaments is of extreme importance for correct ligament-tension-balanced rotational alignment of the femoral component. We note that a weakened medial collateral ligament would lead to an internal rotational alignment. Conversely, a weakened lateral collateral ligament or an insufficient popliteus tendon would result in an external rotational alignment of the femoral component [27]. A balanced flexion gap would still result in these cases. In severe cases, however, a resulting clinically relevant “patellar mal-tracking”, i.e. an unstable patella tracking, should be assumed. Anamnestic and preoperative clinical evaluation of collateral ligament stability is therefore advisable before using a ligament-spanning technique.

Our study has limitations such as the small number of cadavers that could be included in the study as well as the lack of clinical control of the surgical results on living patients. Since the body donors were stored at 4 °C or briefly at -20 °C and no relevant changes were assumed concerning the elasticity of the periarticular soft tissues.

However, an exact determination of the respective axes, due to artifacts caused by the implanted components, posed a problem in previous studies as well as in ours. We realized also as problematic that a uniform classification of the axis designation is not yet established and thus, the axes are designated differently in the literature. It was also not further investigated whether additional ligament compensation was required when the other replacement axes were used for the positioning of the femoral component. It should be noted that all surrogate axes were only determined on axial CT slices, which therefore represent rotation in the sagittal plane. However, the axes could also be angulated in the frontal plane; this was not investigated either preoperatively or postoperatively.

## Conclusion

Our study compares anatomical axes and a navigated functional rotation axis through ligament tension for femoral rotational component alignment in knee arthroplasty with respect to the flexion-extension axis (FEA). This aspect of TKA is still a current issue because the malpositioning of the femoral prosthetic component is a relevant cause of implant failure or clinical failure after TKA. We observed that the determined axes have a significant absolute mean angular deviation from the FEA. For this reason, after analyzing the data from this study, none of the axes examined here can be generally recommended for intraoperative determination of femoral rotation in clinical use. This is also confirmed by the fact that the Cohen’s Kappa of the axis comparisons as a measure of the precision of the values showed only low or moderate correlations. Therefore, surgeons using the anatomical axes for determining femoral rotation should be aware of the fact that potentially clinically relevant incorrect femoral rotation can occur in either internal or external rotation. The presented technique for FEA reconstruction achieves comparable results when compared the use of the known replacement axes, but certain deviations in terms of outliers in internal or external rotation could be observed. The results concerning the fRA has the advantage that a ligament balanced flexion gap without additional ligament releases can be achieved. Thus, an accurate and reliable measurement of fRA appears to be relevant to avoid malpositioning of the femoral prosthetic component which represents the cause of implant failure in the future.

### Electronic supplementary material

Below is the link to the electronic supplementary material.

Supplementary Material 1

## Data Availability

The data that support the findings of this study are available on request from the corresponding author. The data are not publicly available due to privacy or ethical restrictions.

## Abbreviations

aTEA: Anatomical transepicondylar axis
CT: Computer tomography
FEA: Flexion-extension axis
fRA: Functional rotation axis
KA: K-wire axis
N: Newton
PCA: Posterior condylar axis
sTEA: Surgical transepicondylar axis
TKA: Total knee arthroplasty
