# Supplementary figures and images for: Comparison of anatomic axes with a navigated functional rotation axis determined by ligament tension for rotational femoral component alignment in cadaver knee arthroplasty
Source: Arch Orthop Trauma Surg. 2024 Jun 7;144(7):2955–65. doi: 10.1007/s00402-024-05394-3 (PMC11319507; doi:10.1007/s00402-024-05394-3)

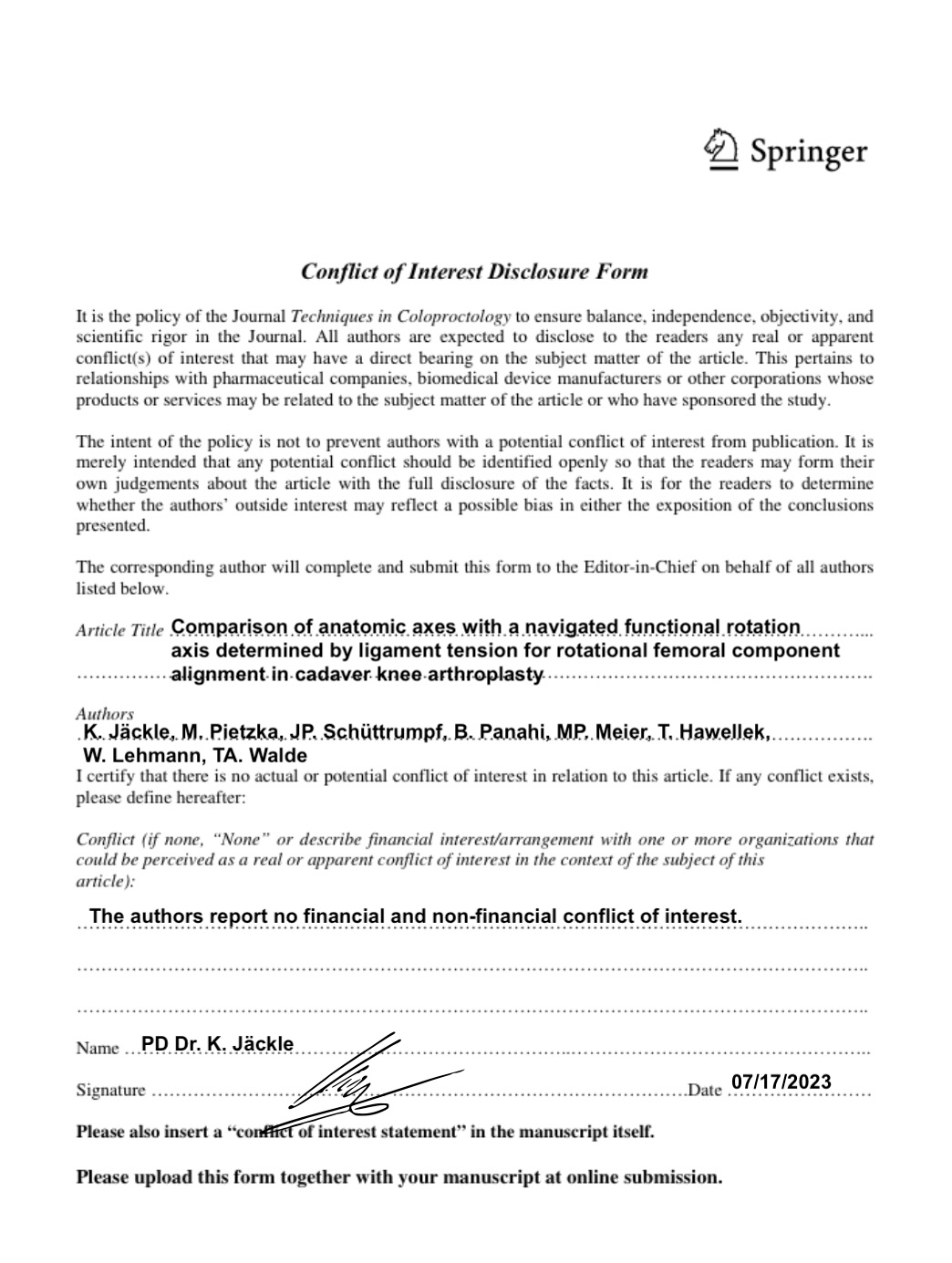

Supplement: Supplementary file 1 — Supplementary Material 1 [file 402_2024_5394_MOESM1_ESM.jpg]
